# Recruitment to a trial of antipsychotic reduction: impact of an acceptability study

**DOI:** 10.1186/s12874-023-01881-0

**Published:** 2023-03-29

**Authors:** Georgina Ramsay, Zoë Haime, Nadia E Crellin, Jacki L Stansfeld, Stefan Priebe, Maria Long, Joanna Moncrieff

**Affiliations:** 1 Research and Development, North East London NHS Foundation Trust, Ilford, Essex UK; 2 University College London, Bloomsbury , UK; 3 Unit for Social and Community Psychiatry, Queen Mary University of London, Mile End, East London, UK; 4 Nuffield Trust, East London, UK

**Keywords:** Randomised controlled trials, RCT, Recruitment, Acceptability study, Schizophrenia, Psychotic disorders

## Abstract

**Objectives:**

Pre-trial acceptability studies may boost recruitment, especially in trials comparing distinctly different interventions. We evaluated the impact of an acceptability study on recruitment to a randomised trial of antipsychotic reduction versus maintenance treatment and explored demographic and clinical predictors of subsequent enrolment.

**Methods:**

Participants with a diagnosis of a schizophrenia spectrum disorder who were taking antipsychotic medication were interviewed about their views of taking part in a future trial.

**Results:**

In a sample of 210 participants, 151 (71.9%) expressed an interest in taking part in the future trial, 16 (7.6%) said they might be interested, and 43 (20.5%) said they were not. Altruistic reasons were most commonly given for wanting to take part, and concern about randomisation for not wanting to. Ultimately 57 people enrolled in the trial (27.1% of the original sample). Eighty-five people who initially expressed an interest did not enrol due to declining or not being eligible (for clinical reasons). Women and people from a white ethnic background were more likely to enrol in the trial, but no illness or treatment-related characteristics were associated with enrolment.

**Conclusion:**

An acceptability study can be a useful tool for recruitment to challenging trials, but it may over-estimate recruitment.

## Introduction

Randomised controlled trials (RCTs) are necessary for producing evidence-based interventions across medicine, including mental health services. Recruitment is often challenging, however [1, 2]. Trials typically require large sample sizes for adequate power but have limited time to recruit due to funding constraints; hence many trials fail to reach their recruitment targets [3], require time extensions to recruit [4] or have to close due to insufficient participant numbers [5].

Barriers to recruitment include organisational difficulties, patient and clinician attitudes, lack of eligible patients, and difficulties in communicating the nature of the research [6–8]. The recruitment of people with serious mental illness, including schizophrenia and other forms of psychosis, presents particular challenges and may be even more difficult [9–11]. Barriers to recruitment include study burden, illness severity, and reluctance to accept diagnosis and treatment [10, 12, 13]. Other factors relate to clinicians and carers, whose involvement is often required in the recruitment process. For clinicians these include a lack of understanding of the research time constraints, high workload; paternalism, including the view that individuals are too unwell to participate in research [14, 15], and stereotypes about certain disorders and their prognosis [16]. Carers may be concerned about research due to poor relationships with clinicians and perceptions that the patient might be upset by the research [17].

Trials that involve a comparison between very different interventions may be particularly challenging to recruit to [7, 18, 19]. Research in other areas, such as surgery, has shown that patients and clinicians often have strong preconceptions about the outcome of different interventions and preferences for one over another, and that communicating clinical equipoise can be challenging [19]. Antipsychotic medication evokes powerful emotions and responses. It is believed to be highly effective in preventing relapse [20], but many patients find it unpleasant to take and it is recognised to produce many adverse effects and serious complications [21, 22].

Acceptability studies offer the opportunity to explore the views of potential participants about proposed interventions and taking part in randomised trials. They may help recruitment by identifying people who might be willing to enrol in advance of the trial starting. Such studies often take the form of interviews or focus groups, typically exploring how a proposed intervention may be received by its target population [23]. If conducted prior to a planned research trial, they can also be utilised to assess the level of interest of potential participants in taking part; understand what barriers to participation there may be and how these could be overcome; and enable the projection of realistic recruitment targets and timeframes. Such studies may be particularly useful for trials that are difficult to recruit to because they involve distinctly different intervention arms. Involving people from the trial target population in an acceptability study also presents a valuable opportunity to invite them to participate in the trial once it begins, as well as to establish links with services, clinicians and carers, thus supporting the development of recruitment pathways. This can help to recruit participants more rapidly and efficiently, increasing the chance of the trial successfully reaching completion.

### Study context

The Research into Antipsychotic Discontinuation and Reduction (RADAR) trial was a randomised trial that compared a gradual, supported process of antipsychotic reduction and discontinuation with maintenance treatment in people with schizophrenia and other psychotic disorders. The primary outcome was social functioning, and severe relapse (defined as admission to a psychiatric inpatient unit) was also evaluated, among other outcomes [24]. The initial part of the trial lasted two years. Prior to the commencement of the trial, a preliminary, interview-based study was conducted to assess the acceptability of the proposed intervention and trial. Those who participated in this preliminary study and expressed interest in taking part in the RADAR trial were later contacted and assessed for eligibility and willingness to enrol.

### Aims

In the current paper, we report data on the effectiveness of the acceptability study in identifying people who could be recruited to the randomised trial. The study aimed to explore people’s views about taking part in a randomised trial of antipsychotic reduction versus maintenance treatment. We also explored differences between those who were ultimately recruited to the trial and those who were not to see if there are features that distinguish people who are more likely to take part in a randomised trial of this sort.

## Methods

### Participants and setting

Participants were recruited from community mental health services and primary care practices across four different areas of London between 2016 and 2017. Participants from primary care settings were not included in the current analysis, as the final trial only recruited from specialist mental health services.

Eligibility criteria for the acceptability study were designed to be as close as possible to those envisaged for the trial. Thus participants had to: (1) have a diagnosis of schizophrenia, schizoaffective disorder, delusional disorder or other non-affective psychotic disorder (i.e. excluding bipolar disorder and psychotic depression); (2) have a history of more than one episode, or a single episode that lasted more than a year, (3) been taking antipsychotics; (4) been stable for a period of at least three months (e.g. not requiring acute care by a crisis team or in an inpatient unit). Exclusion criteria were: (1) lacking of capacity to consent to the research; (2) being legally compelled to take antipsychotic medication; (3) having the potential to present a serious risk of harm to self or others in the view of a treating clinician; (4) requiring an English language interpreter. The eligibility criteria for the subsequent RADAR trial were the same except that the minimum period of clinical stability required prior to recruitment was reduced to one month, and an additional exclusion criterion specified that individuals could not take part if they were pregnant or breast-feeding.

Clinicians were asked to identify patients on their caseload who might meet the eligibility criteria. They explained the study to patients with the support of an information leaflet. Those who agreed were contacted by the research team to arrange a face-to-face interview at a convenient location. Written informed consent was obtained from all participants prior to the interview. Participants were reimbursed for their time and travel.

### Data collection

An interview schedule was designed to elicit participants’ views on long-term antipsychotic medication use and their interest in participating in a future trial to compare a gradual, supported reduction of antipsychotic medication to maintenance treatment (the RADAR trial). The interview schedule was further developed by members of a Lived Experience Advisory Panel (LEAP), consisting of service users and carers. During the interview participants received written and verbal information about the nature and designs of the proposed trial, and were given an opportunity to ask questions about it. They were then asked, “Would you consider entering a trial like this?”. They were also asked about the reasons for their response using some pre-worded options and an option for specifying another reason.

Participants answered questions on demographics, contact with mental health services, their use of antipsychotics and also completed the Drug Attitude Inventory (DAI) [25]. The DAI is a 10-item inventory that assesses attitudes towards taking medication for mental health problems and is scored between 10 (positive attitude) and − 10 (negative attitude).

When the RADAR trial later opened for recruitment, participants who had previously consented to be contacted again were invited to participate in the trial. The numbers of those who did or did not subsequently take part in the RADAR trial were recorded. Reasons for not enrolling were recorded as either ineligible (i.e. did not meet RADAR trial criteria at the time of recruitment) or declined.

### Analysis

All analyses were conducted using Statistical Package for Social Sciences (SPSS, Version 27.0, IBM Corp, 2020). The numbers and proportions of patients who indicated a willingness to participate in the future trial, their reasons for wanting or not wanting to take part and the numbers who actually enrolled were all computed. Differences in potential explanatory variables between people who enrolled in the trial eventually and those who did not were explored using appropriate univariate tests. The following variables were analysed on the basis that they have been shown to influence recruitment decisions in other research or could theoretically impact on motivation to participate in a trial such as RADAR: age, gender, marital status, ethnicity, employment, diagnosis, duration of antipsychotic treatment, form of antipsychotic (long-acting injection (LAI) versus oral) and DAI total score. Logistic regression was used to test the influence of multiple potential predictors that showed at least trend level statistical significance (p < 0.1) in univariate tests. The analysis was exploratory in nature since the sample size was relatively small.

## Results

A total of 210 participants from specialist mental health services were included in the analyses. Table 1 shows the characteristics of the sample according to whether or not they enrolled in the subsequent trial. The majority of the total sample was male (N = 136; 65.1%) with an average age of 45.3 years (SD = 11.24; range 21 to 73). The majority of participants were single (N = 146; 70.2%), three quarters were unemployed (N = 157; 74.8%) and just over half were classified as being from a white ethnic background (N = 113; 53.8%). Most participants were diagnosed with schizophrenia (N = 149; 71.6%). More than half the sample (53.8%) had been in contact with mental health services for more than 15 years and on average they had been taking antipsychotics for 17.0 years (SD = 10.7; range 1 to 49 years). The average total score on the DAI was 2.6 (SD = 5.1, range − 8 to 10).

**Table 1:** Sample characteristics and differences between those who took part in the RADAR trial and those who did not

	Total N = 210	Took Part in RADAR trial N = 57 N (%)	Did Not Take Part in RADAR trial N = 153 N (%)	p-value
**Gender**				0.047
Male	136 (65.1)	31 (54.4)	105 (69.1)	
Female	73 (34.9)	26 (45.6)	47 (30.9)	
**Marital Status (N = 208)**				0.655
Single/Unmarried	146 (70.2)	38 (67.9)	108 (71.1)	
Married/Civil Partnership/Long-term Relationship/Other	62 (29.8)	18 (32.1)	44 (28.9)	
**Ethnicity**				0.049
White	113 (53.8)	37 (64.9)	76 (49.7)	
Non-White/Other	97 (46.2)	20 (35.1)	77 (50.3)	
**Employment**				0.772
Employed (inc. students)	25 (11.9)	7 (12.3)	18 (11.7)	
Unemployed (inc. volunteers)	185 ((88.1)	50 (87.7)	135 (88.3)	
**Diagnosis (N = 208)**				0.547
Schizophrenia	149 (71.6)	41 (71.9)	108 (71.5)	
Other Psychoses	59 (28.1)	16 (28.1)	43 (28.5)	
**Age of MH Diagnosis (N = 208)**				0.326
Less than or equal to 20 years	51 (24.5)	12 (21.4)	39 (25.7)	
21–30 years	99 (47.6)	25 (44.6)	74 (48.7)	
31–40 years	38 (18.3)	13 (23.2)	25 (16.4)	
> 40 years	20 (9.6)	6 (10.7)	14 (9.2)	
**Time in contact with mental health services (N = 208)**				0.212
< 1 year	1 (0.5)	0 (0.0)	1 (0.7)	
1–3 years	11 (5.3)	3 (5.4)	8 (5.3)	
4–10 years	47 (22.6)	13 (23.2)	34 (22.4)	
11–15 years	37 (17.8)	8 (14.3)	29 (19.1)	
16–20 years	30 (14.4)	7 (12.5)	23 (15.1)	
> 20 years	82 (39.4)	25 (44.6)	57 (37.5)	
**Type of Antipsychotic Medication (N = 207)**				0.141
First generation only	72 (34.8)	20 (35.1)	52 (34.7)	
Second generation only (excluding clozapine)	89 (43.0)	29 (50.9)	60 (40.0)	
Clozapine only	28 (13.5)	6 (10.5)	22 (14.7)	
More than one antipsychotic	22 (10.6)	8 (3.9)	14 (6.8)	
**Administration of Antipsychotic Medication (N = 206)**				0.907
Tablet	89 (43.2)	25 (43.9)	64 (43.0)	
Depot	97 (47.1)	30 (52.6)	67 (45.0)	
Both	20 (9.7)	2 (3.5)	18 (12.1)	
		**Mean (SD)**	**Mean (SD)**	
**Age**	45.3 (11.2)	47.0 (12.4)	44.7 (10.8)	0.211
**Time Taking Any Antipsychotic**	17.0 (10.7)	17.9 (11.1)	16.6 (10.6)	0.496
**DAI Total**	2.6 (5.1)	2.3 (5.2)	2.7 (5.1)	0.657

When asked about whether they might be willing to take part in a future randomised trial of antipsychotic medication reduction versus maintenance treatment, 151 (71.9%) said they would, 16 (7.6%) indicated they might and 43 (20.5%) said they would not.

The reasons people endorsed for whether or not they were interested in taking part in the trial are shown in Table 2. The most commonly endorsed reasons for wanting to take part were altruistic, with 52.3% of respondents wanting to help improve treatment for other people and 49.5% saying they wanted to help with research. 40.5% wanted to take part in order to have an opportunity to reduce medication and 40% to reduce the side effects of their medication. A smaller proportion (21.0%) wanted to have more regular appointments with their psychiatrist.

**Table 2:** Reasons for wanting to and not wanting to take part in the trial

	All participants N (%) (N = 210)*
Reasons for wanting to take part	
Improve treatment for others	110 (52.3)
Help with research	104 (49.5)
Opportunity to reduce or stop medication	85 (40.5)
Reduce side effects of medication	84 (40.0)
More psychiatrist appointments	44 (21.0)
**Reasons for not wanting to take part**	
Do not want to reduce or stop medication	34 (16.2)
Concern about randomisation	32 (15.2)
Inconvenience	19 (9.1)
Want to reduce or stop medication	10 (4.8)
Other reasons: concern about relapse, loss of benefits	10 (4.8)

*people could give more than one reason, therefore percentages do not add up to 100.

The most common reasons for not wanting to take part involved not wanting to be randomised (15%), or relatedly having a strong preference either for maintenance treatment (16%) or for antipsychotic reduction (5%).

**Fig. 1:**
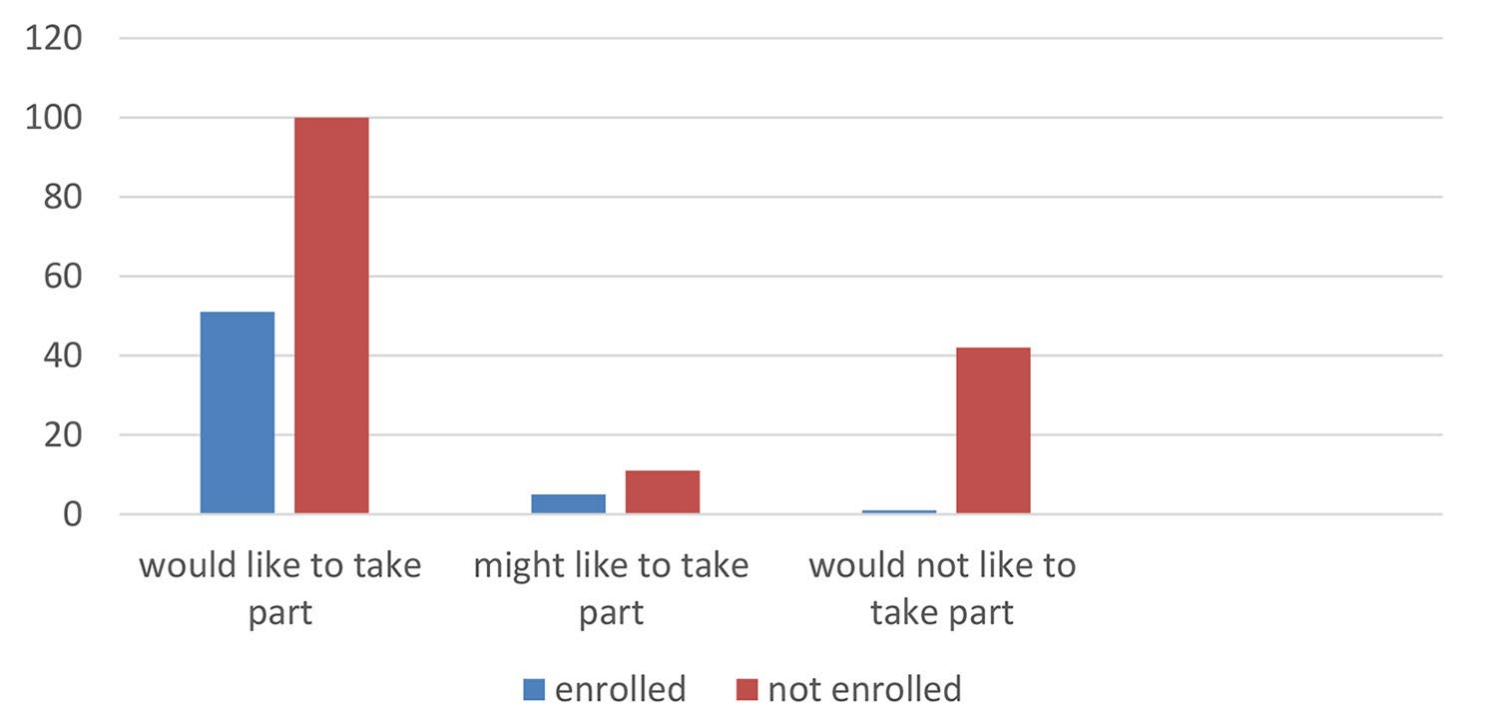
Bar chart of numbers eventually enrolled in the randomised trial by stated willingness in the acceptability study

At least 57 people, representing 27.1% of all those who took part in the acceptability study, ultimately enrolled in the randomised trial. Of those who said they would like to take part, 51 (33.7%) enrolled in the trial. Five people who said they might like to take part were enrolled ( 31.3%) and one person who said they did not want to participate changed their mind and enrolled (Fig. 1). Out of the 151 people who indicated they were willing to take part in the RADAR trial 85 (56.3%) did not end up enrolling and data on enrolment was missing for 15 participants due to inaccessible files from sites external to the main study centre. Of the 85 who were not enrolled, 31 (38.3%) changed their minds and declined participation, and 50 (61.7%) did not meet eligibility at the time of recruitment to the trial. Data on reasons for not enrolling in the trial were missing for four participants.

In univariate tests, there were no differences between people who subsequently enrolled in the trial and those who did not in demographic or clinical characteristics, or in attitudes towards antipsychotic medication as measured by the DAI (Table 1). However, there were trend level differences in gender and ethnicity, with people who subsequently enrolled in the trial being more likely to be female and from a white ethnic group. Logistic regression analyses including gender and ethnicity as potential predictors showed a statistically significant effect on likelihood of being recruited to the trial (Table 3). Women were 2.0 times more likely to take part than men and people classed as ‘white’ were 1.9 times more likely to take part than those of a non-white ethnicity.

**Table 3:** Logistic regression of predictors of enrolling in the randomised trial

	B	Standard Error	Wald	Degrees of Freedom	p value	Exp (B)	95% CI for Exp (B)
Gender (male vs. female)	0.68	0.32	4.4	1	0.037	0.51	0.27–0.96
Ethnicity (white vs. non-white)	0.62	0.33	4.1	1	0.042	1.94	1.0-3.7
Constant	0.94	0.31	9.5	1	0.002	0.39	

## Discussion

### Key findings

Ultimately, over a quarter of people (27.1%) who participated in the acceptability study enrolled in the randomized trial of antipsychotic reduction. This was fewer than the 71.9% of participants who said they would consider taking part when they were interviewed, but nevertheless it represented a substantial number of people who were recruited to the trial quickly and easily once it was opened. This is particularly valuable in a trial involving starkly different treatment strategies, since such trials may be particularly difficult to recruit to [7, 18, 19].

People’s reasons for wanting to take part in the study reflect other research in finding that altruism was a common motivation [26, 27], and also wanting access to the experimental intervention, [28], in this case the supported antipsychotic reduction programme. People also commonly identified access to more frequent appointments as a reason for wanting to take part in the trial. This was unexpected and may reflect a mismatch between the standard of care currently provided in mental health services and the expectations of people with long term psychiatric conditions. Reasons for not wanting to take part mirror other research in that many people found randomization unacceptable and/or expressed a strong preference for one type of treatment over the other [26, 29].

Of those who indicated an interest in taking part in the trial, 56% did not end up participating, either because they declined or were not considered eligible. Although we do not know the precise reasons why people were not eligible, this is a high number, given that the eligibility criteria for the trial were almost identical to those for the acceptability study. Some people may have become mentally unwell between taking part in the acceptability study and being approached about the trial, and therefore not satisfied the requirement to be mentally stable. Another potential reason is different judgements about risk. For both studies, an exclusion criteria was if the clinician judged that the participant posed ‘a serious risk of harm to self or others’. This criteria is likely to have been applied more leniently in the acceptability study, which only involved an interview, than in the randomised trial that involved the possibility of having antipsychotic medication reduced and discontinued.

The number of people who declined to enter the trial after having expressed an interest in doing so may reflect changes in circumstances, or that people’s decision-making changes when confronted with the reality, rather than only the theoretical possibility, of entering a trial of this sort. Other data from this acceptability study suggests that fear of relapse was a prevalent reason why people did not want to reduce or discontinue their antipsychotic medication [30], and this has also been found to be an important concern among patients and carers in other research [31, 32]. Considering that participants in this study had been taking antipsychotics for almost 17 years on average, with an average drug attitude index score trending towards positive, it is likely that many potential participants were dissuaded from taking part in the trial by the possible increased risk of relapse. This concern may have become more focused when confronted with the realities of taking part in the trial. Alternatively, it is possible that some people’s responses to the acceptability study questions were influenced by social desirability bias and that they never really wanted to take part in the first place but felt compelled to express an interest in doing so.

The finding that women were more likely to be recruited to the trial than men contrasts with other antipsychotic trials in which women are generally under-represented [33]. Our finding that people from ethnic minority backgrounds were less likely to be recruited to the trial is consistent with other research on recruitment to studies involving people with mental health problems [34] and other conditions [35]. It is notable that no clinical or treatment characteristics predicted recruitment, including people’s attitudes to their antipsychotic medication. This suggests the final trial sample will not over-represent people with particular clinical characteristics in comparison with the acceptability study. Previous research has found that people with psychosis are less likely to be approached for studies if they are more severely unwell [15], therefore, people in both the acceptability study and the trial may be less severely ill than an average sample of people with a psychotic diagnosis. However, a study of a subsample of the RADAR trial participants found that indicators of severity did not differ when compared to those of people with eligible diagnoses from the same mental health service who were not recruited [36].

Implications for research.

Running an acceptability study can provide valuable information to help understand recruitment to a randomised trial, including potential barriers to participation and characteristics that may make it more difficult to recruit particular groups. This enables the construction of a detailed trial recruitment strategy. This might include adapting the language used to explain the need for the trial, including the concept of clinical equipoise, the process of randomisation and the intervention itself to make it more accessible and avoid misunderstandings. Methods to target people from ethnic minority backgrounds can also be devised, including the use of interpreters when resources allow. However, the fact that there were no clinical predictors of who would ultimately enroll means there is no obvious way of targeting recruitment and recruitment efforts need to be broad and inclusive.

An acceptability study also allows researchers to access a sample of people who are likely to be eligible and interested in participating as soon as the recruitment stage of the trial opens, enabling more rapid recruitment. Participation in an acceptability study can improve patient familiarity with research processes in general, and by providing advanced information about the trial, can make it a less intimidating decision to agree to take part. If the individual researchers conducting the acceptability study and trial recruitment can be kept the same, as they were in many instances in the current study, then this establishes rapport and provides further reassurance, thereby improving the chances of recruitment to the trial. Experience from other trials involving people with psychosis has shown that building relationships between researchers participants, as well as clinicians, helps with recruitment [37].

Despite its potential usefulness, the relatively large proportion of people who expressed interest in participating in the trial but then did not take part (56%) indicates that this sort of acceptability study can over-estimate rates of trial participation, which needs to be factored into recruitment estimations and planning.

Limitations.

The data in the current study was gathered in reference to a particular trial. Levels of recruitment from an acceptability study are likely to vary according to the nature of the trial and the interventions involved. The RADAR trial involved strongly contrasting intervention and control conditions and reasons that people gave for not wanting to take part included a preference for the intervention (antipsychotic reduction) and a preference for the control (maintenance antipsychotic treatment) arm. Trials that offer less starkly contrasting treatments, or those in which the intervention represents an addition to usual care, may not have the same challenges.

The RADAR trial also required clinicians to make a decision about one of the eligibility criteria (presenting a risk of harm to oneself or others). Although the eligibility criteria of the acceptability study were intended to match those of the randomized trial, it is likely that this criterion was applied differently, resulting in a lower proportion of people being considered eligible for the trial than anticipated. Unfortunately, we did not have details about the precise reasons why people were not considered eligible for the trial, so it is difficult to confirm this suspicion.

## Conclusion

An acceptability study involving a single, semi-structured interview enabled the recruitment of over a quarter of the participants into a subsequent randomized trial of antipsychotic reduction. Given the challenges of recruitment in trials involving people with severe mental health problems in particular, acceptability studies can be a valuable investment. There were no clinical predictors of subsequent recruitment to the trial, suggesting that recruitment efforts need to be as inclusive as possible.

## Data Availability

The datasets generated and/or analysed during the current study are not publicly available but may be available from the corresponding author on reasonable request.

## Abbreviations

RCT: Randomised controlled trial
RADAR: Research into Antipsychotic Discontinuation and Reduction
LEAP: Lived Experience Advisory Panel
DAI: Drug Attitude Inventory
LAI: Long-acting injection
